# Estimation of Influenza Vaccine Effectiveness from Routine Surveillance Data

**DOI:** 10.1371/journal.pone.0005079

**Published:** 2009-03-31

**Authors:** Heath Kelly, Kylie Carville, Kristina Grant, Peter Jacoby, Thomas Tran, Ian Barr

**Affiliations:** 1 Epidemiology Unit and Virus Identification Laboratory, Victorian Infectious Diseases Reference Laboratory, Melbourne, Australia; 2 Telethon Institute for Child Health Research, Centre for Child Health Research, the University of Western Australia, Perth, Australia; 3 World Health Organization Collaborating Centre for Reference and Research on Influenza, Melbourne, Australia; Yale University, United States of America

## Abstract

**Background:**

Influenza vaccines are reviewed each year, and often changed, in an effort to maintain their effectiveness against drifted influenza viruses. There is however no regular review of influenza vaccine effectiveness during, or at the end of, Australian influenza seasons. It is possible to use a case control method to estimate vaccine effectiveness from surveillance data when all patients in a surveillance system are tested for influenza and their vaccination status is known.

**Methodology/Principal Findings:**

Influenza-like illness (ILI) surveillance is conducted during the influenza season in sentinel general practices scattered throughout Victoria, Australia. Over five seasons 2003–7, data on age, sex and vaccination status were collected and nose and throat swabs were offered to patients presenting within three days of the onset of their symptoms. Swabs were tested using a reverse transcriptase polymerase chain reaction (RT-PCR) test. Those positive for influenza were sent to the World Health Organization (WHO) Collaborating Centre for Reference and Research on Influenza where influenza virus culture and strain identification was attempted. We used a retrospective case control design in five consecutive influenza seasons, and estimated influenza vaccine effectiveness (VE) for patients of all ages to be 53% (95% CI 38–64), but 41% (95% CI 19–57) adjusted for age group and year. The adjusted VE for all adults aged at least 20 years, the age groups for whom a benefit of vaccination could be shown, was 51% (95% CI 34–63). Comparison of VE estimates with vaccine and circulating strain matches across the years did not reveal any significant differences.

**Conclusions/Significance:**

These estimates support other field studies of influenza vaccine effectiveness, given that theoretical considerations suggest that these values may underestimate true effectiveness, depending on test specificity and the ratio of the influenza ILI attack rate to the non-influenza ILI attack rate. Incomplete recording of vaccination status and under-representation of children in patients from whom a swab was collected limit the data. Improvements have been implemented for prospective studies.

## Introduction

Influenza vaccination is required each year because the predominant circulating strains of the influenza virus drift over time. The composition of influenza vaccines is reviewed annually and vaccine constituents are often changed in an effort to maintain protection against drifted influenza virus strains. In temperate southern Australia, the influenza season occurs between about May (late autumn) and October (early spring). The Australian Influenza Vaccine Committee meets in October of the preceding year to choose the influenza strains that will be included in the vaccine. Influenza vaccine is usually available by February or March of the following year, at least two months, but usually closer to four months, before the influenza season. Influenza vaccine is provided free of cost to all adults aged 65 years and over and adults of Aboriginal and Torres Island descent aged 50 years and over and is recommended, but not funded, for other groups.

Most countries where influenza vaccine is offered as part of a publicly funded program do not routinely monitor the success of vaccine strain selection by monitoring seasonal influenza vaccine effectiveness (VE). We use the conventional definitions of vaccine effectiveness as an estimate from an observational study whereas vaccine efficacy is an estimate derived from a trial. Vaccine efficacy is defined as the percentage reduction of cases among vaccinated individuals. Where VE is monitored, it is most efficiently done using routinely collected data available from sentinel surveillance networks.[1], [2] These VE estimates can be compared with estimates of efficacy against clinical disease due to laboratory confirmed influenza from trials and meta-analyses. Published efficacy estimates range from 63% (95% CI 45–70) for children [3] to 74% (95% CI 55–81) for healthy young adults [4] and 58% (95% CI 26–77) for people aged 60 years and over from a large randomised controlled trial.[5] However a Cochrane review concluded that influenza vaccine was not efficacious against laboratory confirmed influenza in the elderly.[6]

France is unique in using the screening method to monitor influenza VE as the influenza season unfolds. This allows early recognition of possible poor vaccine effectiveness.[1] However, to estimate VE, the screening method requires accurate assessment of a relatively high vaccination coverage.[7] Often neither of these conditions is met. It has recently been demonstrated that it is possible to estimate VE from routine surveillance data using all patients with an influenza-like illness (ILI) who have presented for medical attention and have subsequently been tested for influenza.[8] VE is estimated as 1−OR, where OR is the odds ratio from a case control study. Cases and controls are selected from the cohort of all patients recruited from an influenza sentinel surveillance network who present with an ILI. All patients with an ILI who have been tested for influenza using a standard laboratory assay are eligible for inclusion in the study. Cases are ILI patients with laboratory confirmed influenza and controls are ILI patients without laboratory confirmed influenza. Controls may have another respiratory virus or no virus detected. We aimed to perform a retrospective estimate of influenza VE against medically attended laboratory confirmed influenza for five influenza seasons between 2003 and 2007 in Victoria, Australia. We used the ILI case control design and data collected routinely from seasonal sentinel surveillance. We also compared annual estimates of VE with a review of the match between the circulating and vaccine strains for each season.

## Materials and Methods

### Routine sentinel surveillance

Victoria is a southern Australian state with a temperate climate. The influenza season occurs in winter and often extends into the early months of spring. Between May and September each year, sentinel surveillance is conducted in general practices scattered throughout Melbourne and regional Victoria. Victoria's population is approaching 5 million, with 3.85 million people living in the state capital, Melbourne. Laboratory supported surveillance began in 1998 [9] and surveillance reports are published annually (available at http://www.cdc.gov/ncidod/eid/vol1no1/longbotm.htm). For each season, participating general practitioners (GPs) were asked to report weekly on the total number of consultations and any patients presenting with ILI, defined as history of fever, cough and fatigue/malaise.[10] Once formal consent was obtained from these patients, GPs collected data on their age, sex, symptoms and vaccination status. Nose and throat swabs were offered to patients presenting within three days of the onset of their symptoms. Swabs, pooled in a single vial of viral transport medium, were transported by courier to the Victorian Infectious Diseases Reference Laboratory (VIDRL) where they were tested using an in-house respiratory multiplex reverse transcriptase polymerase chain reaction (RT-PCR) test identifying influenza viruses, adenoviruses, picornaviruses (enteroviruses and rhinoviruses), respiratory syncytial virus and parainfluenza viruses.[11] All nose and throat swabs from sentinel ILI patients were stored at −70°C and those that were positive for influenza were transported frozen to the World Health Organization (WHO) Collaborating Centre for Reference and Research on Influenza where influenza virus culture and strain identification was attempted.

Written consent was obtained from patients at the time a swab was taken, indicating that aggregate anonymous data will be used for surveillance purposes and influenza positive results will be notified to the state government Department of Human Services, Victoria. Laboratory confirmed influenza has been a gazetted notifiable disease in Victoria since 2001. Because of the legal requirement for the laboratory to notify positive cases, we have been advised by the Victorian Department of Human Services that formal ethics approval is not required for the surveillance program.

Seasonal thresholds were based on rates of ILI cases per 1000 consultations. Baseline activity, normal seasonal and higher than expected seasonal activity were defined as below 2.5, between 2.5 and <15, and between 15 and <35 per 1000 consultations, respectively. According to these thresholds, ‘epidemic influenza activity’ was defined by rates at or above 35 cases per 1000 consultations.[12]

### Estimation of VE

We restricted our analysis to patients who presented for medical attention at any of the sentinel surveillance sites and who subsequently had a swab taken for the identification of influenza virus by RT-PCR. Patients whose PCR tests were inhibited were excluded from the analysis, as were patients whose vaccine status or age was unknown. We examined the proportion of cases and controls with unknown vaccination status and also examined unknown vaccination status by age group. Counting all patients whose swabs were positive for influenza virus RNA as cases and all other patients whose swabs were negative or positive for another respiratory virus as controls, we estimated unadjusted VE = 1−OR, where OR is the odds of being a vaccinated case divided by the odds of being a vaccinated control. We report only the OR when there is no apparent protective effect of vaccination. We performed an age-adjusted estimate of VE by logistic regression using the following age groups: 0–4 years, 5–19 years, 20–49 years, 50–64 years and 65 years and above. We also adjusted for year. We examined homogeneity by age group and year using the Mantel-Haenszel test for homogeneity. We tested whether the estimates of VE were significantly different by testing whether the difference between the natural logarithms of the ORs differed from zero, where the standard error (SE) of ln OR1−ln OR2 was calculated as the square root of [SE (ln OR1)^2^+SE (ln OR2)^2^]. Two-sided p-values for the null hypothesis were obtained from the Normal distribution. The 5% significance level was used for all comparisons. Surveillance data were maintained in a purpose designed database (SL Digital) and data were exported from this database to STATA 10 [13] for all statistical analyses.

### Analysis of circulating and vaccine strains

Influenza viruses were received by the WHO Collaborating Centre for Reference and Research on Influenza from the surveillance program at VIDRL and from other laboratories in Melbourne. Viruses were received as isolates, passaged in cell culture, or as original clinical samples in which influenza A or B antigen had been detected by immunofluorescence or RNA detected by RT-PCR. Once received at the Centre, the isolates or samples were cultured in MDCK cells (CCL-34, ATCC, USA) and monitored for growth by cytopathic effect and the presence of haemagglutinating activity using Turkey red blood cells as previously described.[14] Positive samples were typed using the haemagglutination inhibition assay (HI) against a panel of known standard reference viruses (including vaccine strains) and their homologous ferret antisera.[14] Prior to use, ferret antisera were pre-treated with receptor destroying enzyme (RDE) (Denka Seikan, Japan), to remove non-specific inhibitors. Viruses were considered to be vaccine-like if their HI titre was no more than 4-fold lower than the homologous vaccine virus titre. Vaccine strains were those selected by the Australian Influenza Vaccine Committee and approved by the Therapeutics and Goods Administration (TGA) for use in Australia each year (http://www.tga.gov.au/committee/aivc.htm). The proportion of each influenza type/subtype circulating was obtained from the total influenza viruses isolated at the WHO Influenza Centre in Melbourne from Victorian patients in each calendar year.

## Results

### Influenza seasons and ILI patients

The number of GPs participating in sentinel surveillance ranged from 65 to 79 for each of the five influenza seasons and GPs recorded 5,000 ILI consultations from a total of more than 700,000 consultations (Table 1).

**Table 1:** General practitioners, ILI consultations and total consultations, Victorian sentinel influenza surveillance, 2003–2007.

Year	Sentinel practitioners	ILI consultations	Total consultations
2003	79	1283	156,445
2004	76	820	166,626
2005	74	1087	149,018
2006	74	765	136,732
2007	65	1045	120,256

**Figure 1:**
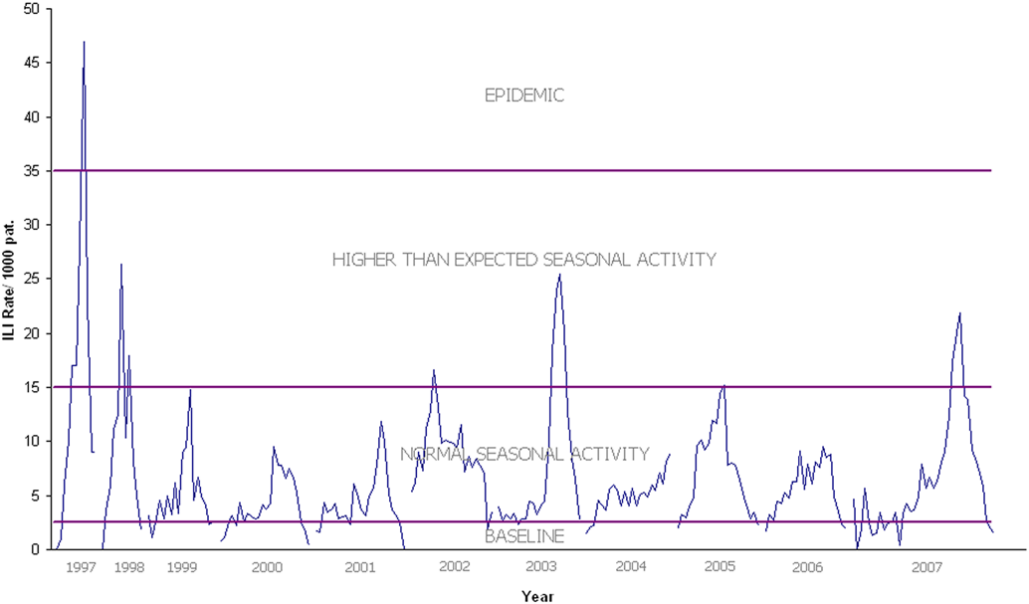
Influenza-like illness 1997 to 2007 from general practice sentinel surveillance.

**Figure 2:**
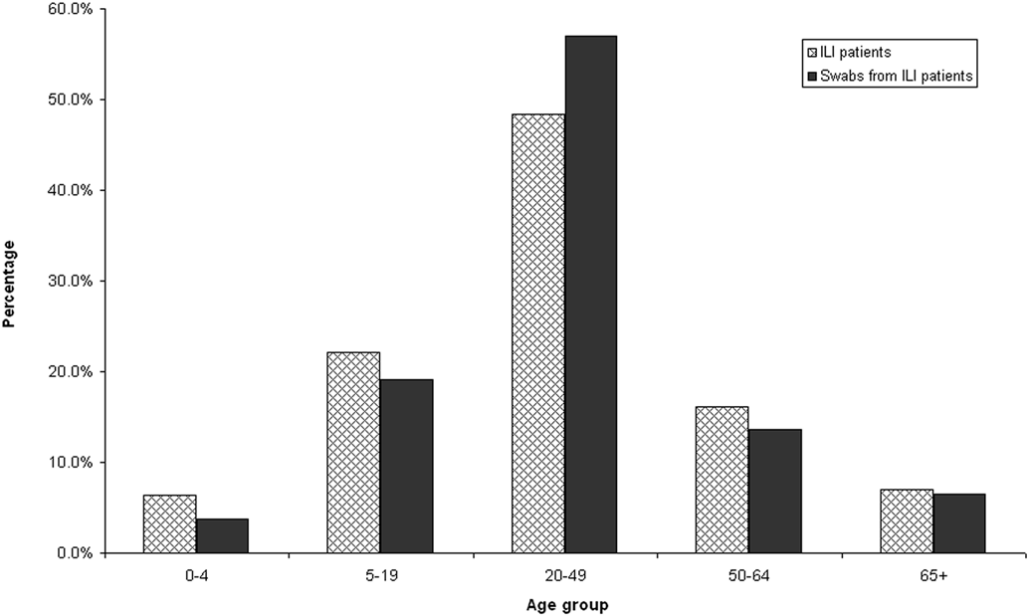
Laboratory confirmed influenza and ILI cases by proportion of age group, 2003–2007.

The five influenza seasons included in the VE assessment were characterised by higher than expected seasonal activity in 2003 and 2007 and normal seasonal activity in the other three years. Earlier surveillance years, not included in the study of VE, have been included for comparison of relative influenza activity (Figure 1). The age distribution of ILI patients for whom a nose and throat swab was collected was similar in all years and is shown, compared with the age distribution of all patients recorded as attending a sentinel practice with an ILI (Figure 2). In all five influenza seasons, 71% of ILI patients were aged 20–64 years. Patients with an ILI aged less than 19 years accounted for 23% of all patients seen and those aged 65 years and above comprised 7%.

Swabs were taken from approximately 40% (2065/5000) of the ILI patients seen by sentinel surveillance GPs. Laboratory results were considered for 2015 patients (15 patients were excluded as age was unknown and 35 were excluded as the PCR test was inhibited). Vaccination status was known for 73% (n = 1479/2015) of the patients with laboratory results, ranging from 65–80% over the five influenza seasons. Influenza virus was detected from an average of 36% (range 18–47) of all ILI patients with a laboratory result over the five seasons. Other viruses accounted for ILI in an average of another 16% (range 10–29) of patients (Table 2).

**Table 2:** Sampled ILI patients with influenza or another respiratory virus detected, by vaccination status, Victoria 2003–2007.

Year	N tested	Vaccinated			Unvaccinated			Unknown		
		n	Influenza detected (% of n)	Other respiratory virus detected (%)	n	Influenza detected (% of n)	Other respiratory virus detected (%)	n	Influenza detected (% of n)	Other respiratory virus detected (%)
2003	535	87	23 (26%)	13 (15%)	263	104 (40%)	22 (8.4%)	185	56 (30%)	21 (11%)
2004	265	61	6 (9.8%)	19 (31%)	137	32 (23%)	37 (27%)	67	10 (15%)	21 (31%)
2005	397	63	18 (29%)	16 (25%)	244	116 (48%)	28 (11%)	90	46 (51%)	18 (20%)
2006	381	54	14 (26%)	9 (17%)	222	81 (36%)	34 (15%)	105	31 (30%)	19 (18%)
2007	437	79	26 (33%)	13 (16%)	269	140 (52%)	35 (13%)	89	38 (43%)	14 (16%)
All	2015	344	87 (25%)	70 (20%)	1135	473 (42%)	156 (14%)	536	181 (34%)	93 (17%)

### VE estimates compared with circulating and vaccine strains

VE estimates were calculated for the 1479 patients for whom age, laboratory results, and vaccination status data were available. The unadjusted estimate of VE for each of the five seasons ranged from 39% (95% CI −19 to 67) to 64% (95% CI 9–86) and, for the five seasons combined, was 53% (95% CI 38–64) (Table 3).

**Table 3:** Estimates of influenza vaccine effectiveness (VE) with vaccine and circulating strains.

Year	Vaccine effectiveness (95% CI)	Age adjusted vaccine effectiveness (95% CI)	Type/subtype	% type/subtype in that year in Victoria	Vaccine strain (or like strain) used/recommended	Most commonly circulating like-strain(s) in Victoria
2003	45 (6 , 68)	40 (−13 , 68)	A(H1)	0	A/New Caledonia/20/99	-
			A(H3)	99.5	A/Moscow/10/99^1^	A/Fujian/411/2002 (H3N2)
			B	0.5	B/Hong Kong/330/2001^2^ *	
						
2004	64 (9 , 86)	52 (−28 , 82)	A(H1)	0	A/New Caledonia/20/99	-
			A(H3)	62.8	A/Fujian/411/2002	A/Fujian/411/2002 (H3N2)
			B	37.2	B/Hong Kong/330/2001^2^ *	B/Shanghai/361/2002+
						
2005	56 (19 , 76)	34 (−33 , 67)	A(H1)	36.8	A/New Caledonia/20/99	A/New Caledonia/20/99 (H1N1)
			A(H3)	44.8	A/Wellington/1/2004	A/Wellington/1/2004 and A/California/7/2004 (H3N2)
			B	18.4	B/Shanghai/361/2002^3^ +	B/Shanghai/361/2002+ and B/Hong Kong/330/2001*
						
2006	39 (−19 , 67)	16 (−77 , 60)	A(H1)	2.1	A/New Caledonia/20/99	-
			A(H3)	67.2	A/California/7/2004^4^	A/Wisconsin/67/2005 (H3N2)
			B	30.7	B/Malaysia/2506/2004*	B/Malaysia/2506/2004
						
2007	55 (23 , 73)	54 (15 , 75)	A(H1)	36.6	A/New Caledonia/20/99	A/Brisbane/59/2007 (H1N1)
			A(H3)	50.0	A/Wisconsin/67/2005	A/Brisbane/10/2007 (H3N2)
			B	13.4	B/Malaysia/2506/2004*	B/Florida/4/2006+

1 Actual vaccine strain used A/Panama/2007/99.

2 Actual vaccine strain used B/Shangdong/7/97 or B/Brisbane/32/2002.

3 Actual vaccine strain used B/Jiangsu/10/2003.

4 Actual vaccine strain used A/New York/55/2004.

* B/Victoria/2/87-lineage.

+ B/Yamagata/16/88-lineage.

Vaccination status was unknown for 27% (536/2015) of patients, 24% (181/741) for cases and 28% (355/1274) for controls (p = 0.1). There was no significant difference in the proportion of patients with unknown vaccination status by age group. Vaccination status was unknown for 34% of 0–4 years, and 25–27% of all other age groups (p = 0.6).

When VE estimates were adjusted for age and year they declined. The age and year adjusted estimate of VE for the five seasons combined was 41% (95% CI 19–57). Homogeneity by sub-group could not be rejected for year (p = 0.89) but lack of homogeneity was suggested by age group (p = 0.07). We therefore report results by age group over the five years. In this period the OR could not be calculated for children aged 0–4 years, as there were no vaccinated children without influenza. This age group comprised only 2% (48/2015) of patients with laboratory testing. There was no apparent protection for older children and young adults aged 5–19 years (OR = 1.6, 95% CI 0.6–4.9). However moderate to good protection was seen for adults aged 20–49 and 50–64 years with VE estimates adjusted for year equal to 42% (95% CI 13–61) and 57% (95% CI 17–78) respectively. For these two age groups combined, the age and year adjusted VE was 46% (95% CI 26–62). In all five seasons, influenza virus was detected in 26/99 patients aged 65 years and over and VE adjusted for year was estimated as 69% (95% CI 8–90). There were no significant differences in VE estimates for any of the three adult age groups. The age group and year adjusted VE for all adults aged at least 20 years was 51% (95% CI 34–63).

Vaccination status and the proportion of patients with laboratory confirmed influenza differed significantly by age group. We compared patients aged 0–19, for whom no benefit of vaccination could be shown, with all older age groups. Only 6% (19/329) of patients aged 0–19 were vaccinated compared with 28% (325/1150) of older patients (p<0.001). For those aged 65 years and over, who were eligible for free vaccine, the proportion vaccinated was 82% (82/99). Of younger patients, 53% (173/329) tested positive for influenza compared with 34% (387/1150) of older patients (p<0.001).

Comparison of VE estimates with vaccine and circulating strains is shown in Table 3 for each of the five influenza seasons. In 2003 more than 99% of all circulating virus strains in Victoria were A/Fujian/411/2002 (H3N2)-like, but the corresponding vaccine strain was A/Moscow/10/99. In that year the age adjusted VE was 40% (95% CI −13 to 68). The following year there was an exact match between the H3 vaccine and circulating strains (Fujian). Although the H3 sub-type comprised only about 63% of circulating strains, the remainder of which were influenza B with mismatch between vaccine and circulating strains, the age adjusted VE estimate in this year was 52% (95% CI −28 to 82). However this estimate was based only on 38 ILI patients with laboratory confirmed influenza (Table 2). In 2005 when circulating strains were spread between influenza H1, H3 and B with good vaccine strain match, influenza VE was estimated as 34% (95% CI −33 to 67). Reflecting some H3 mismatch in 2006, the VE estimate was lower at 16% (95% CI −77 to 60) in a season again characterised by influenza H3 and B. Despite apparent mismatches with H1, H3 and B in 2007, all of which were circulating, the VE estimate was 54% (95% CI 15–75).

## Discussion

Using routinely collected surveillance data, we have shown an age and year adjusted estimate of influenza VE of 41% (95% CI 19–57) over five consecutive seasons for all age groups but 51% (95% CI 34–63) for adults aged at least 20 years, for whom a benefit of vaccination could be shown. In the sample of patients from our surveillance system there was no apparent protective effect for age groups under 19 years and influenza VE estimates were non-significantly higher for adults aged 50–64 and 65 years and over compared with adults aged 20–49 years. We were unable to show any difference in VE in the years when the vaccine strains were well matched compared with years when the match was poorer. A non-significant tendency for VE estimates to be lower in years with an H3 mismatch was seen in 2003 and 2006 but not in 2007.

There are a number of issues that impact on the VE estimates obtained. The small number of patients and low vaccination rates in the younger age groups indicates that our sentinel surveillance system is better suited to estimating VE amongst working age adults who comprised almost three quarters of our surveillance population. Theoretical considerations suggest that the ILI case control method for estimating VE from surveillance data may underestimate true effectiveness, depending on test specificity and the ratio of the influenza ILI attack rate to the non-influenza ILI attack rate.[8] However the specificity of the PCR assay used in this study has been estimated as 100% for influenza A and B [11], so that the major impact of study design on subsequent VE estimate will be the ratio of the influenza ILI attack rate to the non-influenza ILI attack rate. This impact is modeled to be minimal for high test specificity (Figure 3, [8]), so that study design should not have had a major effect on VE estimates. The various mismatches between circulating and vaccine strains may have led to lower than expected estimates of VE.

Age was a confounder in our estimates of VE. Compared to all older age groups, a higher proportion of the sample of children and younger adults aged 0–19 years tested influenza positive but a lower proportion was vaccinated. This resulted in a fall in VE estimates when adjusted for age.

Comprehensive recording of vaccine status remains an issue in our surveillance network but is not likely to have had a significant effect on our estimate of VE. In the Canadian study [2], vaccine status was unknown for only 13/524 (2%) of patients compared with 27% in this study. However we have demonstrated no significant difference in unknown vaccination status by case/control status (outcome) or age group (confounder). Missing vaccination status should therefore have no significant effect on the estimate of VE. Improvements have been implemented in the ascertainment of vaccination status for prospective studies.

Our summary VE estimate is lower than that obtained in Canada for the 2005–6 season when there was a dual A and B mismatch. Using the same study design as reported here, the unadjusted VE estimate for influenza A or B from the Canadian study was 65% (95% CI 42–79), falling to 58% (95% CI 24–76) when adjusted for age but rising again to 61% (95% CI 26–79) when adjusted for age and chronic conditions.[2] Our VE estimate also fell when adjusted for age. We do not routinely collect information on co-morbidities and therefore could not adjust for these conditions.

We did not estimate VE separately for subtype because our subtype data were incomplete over the 5 years. VE estimates by subtype can be used to further evaluate vaccine strain selection but it is the summary VE estimate that is published in meta-analyses [4] and used in studies attempting to establish the cost effectiveness of influenza vaccine.[15], [16] The summary estimate is of most interest to policy makers, although it should be recognised that this estimate attempts to capture VE for influenza B, H1 and H3, circulating in different proportions at different thresholds, and with varying circulating and vaccine strain mismatch in each season.

Using the screening method to estimate field VE, based on data from the French sentinel practice network to estimate the proportion of patients vaccinated and population surveys to estimate the proportion of the population vaccinated, estimates of VE against ILI ranged from 42% (95% CI 31–51) to 76% (95% CI 68–81) for patients aged 15–64 years but were lower for patients aged 65 years and above.[1] In our population estimates of VE against laboratory confirmed influenza were higher in patients aged 65 years and above but we had too few patients aged 65 years and above to report VE by year in this age group. If this difference in VE is not explained by sampling error, an apparent higher VE in older people may represent a more healthy population of ambulatory older people being seen in general practice, with more at-risk patients attending hospitals or being seen by GPs in aged-care facilities. Obtaining co-morbidity data for patients may assist in understanding these findings.

In using surveillance data for research purposes, it would be ideal if sentinel practices were representative of all general practices. We have previously demonstrated that our sentinel network adequately describes ILI activity in Victoria.[17] However, we know that ILI patients from whom a nose and throat swab are taken, are not representative of all ILI patients or of all patients notified with influenza. Children were under-represented in patients from whom a swab was collected in the sentinel practices (Figure 2).

Despite its limitations, we have demonstrated that the Victorian sentinel surveillance network is able to provide estimates of influenza VE. We have further compared VE estimates with vaccine and circulating strain matches and, while there were no significant differences in VE across the years, there was some suggestion that VE may be lower in years when the influenza A/H3N2 subtype is mismatched, perhaps reflecting the fact that infection with the H3N2 subtype is generally more severe in adults.[18], [19], [20] Routine monitoring data of this type will be further interrogated to add value to the sentinel surveillance of influenza.
